# Organ-Sparing Surgery in Testicular Tumor: Is This the Right Approach for Lesions ≤ 20 mm?

**DOI:** 10.3390/jcm9092911

**Published:** 2020-09-09

**Authors:** Nina Staudacher, Gennadi Tulchiner, Katie Bates, Michael Ladurner, Mona Kafka, Friedrich Aigner, Renate Pichler, Wolfgang Horninger

**Affiliations:** 1 Department of Urology, Medical University Innsbruck, Anichstrasse 35, 6020 Innsbruck, Austria; Nina.Staudacher@i-med.ac.at (N.S.); Gennadi.Tulchiner@i-med.ac.at (G.T.); Michael.Ladurner@tirol-kliniken.at (M.L.); Mona.Kafka@i-med.ac.at (M.K.); Wolfgang.Horninger@i-med.ac.at (W.H.); 2 Department of Medical Statistics, Informatics and Health Economics, Medical University Innsbruck, Schoepfstraße 41, 6020 Innsbruck, Austria; Katie.Bates@i-med.ac.at; 3 Department of Radiology, Medical University Innsbruck, Anichstrasse 35, 6020 Innsbruck, Austria; Friedrich.Aigner@tirol-kliniken.at

**Keywords:** testicular cancer, small testicular tumors, organ-sparing surgery, scrotal ultrasonography, frozen section examination

## Abstract

**Background:** This study was conducted in order to analyze factors predicting malignancy in patients undergoing organ-sparing surgery (OSS) for small testicular lesions. **Methods:** Patients with small (≤20 mm) marker-negative clinical stage I testicular tumors were managed by OSS with tumor enucleation and frozen section examination (FSE) for the past 15 years at our institution. Benign and malignant cases were compared, focusing on preoperative and postoperative lesion sizes. **Results:** Eighty-nine patients were enrolled in this retrospective study. Ten (11.2%) of them were treated for synchronous bilateral tumors. Sixty-seven (67.7%) of ninety-nine lesions were benign, confirming a high concordance rate (98%) between FSE and final histology. Patients with benign tumors were significantly older than patients with malignant tumors (*p* = 0.026), and benign tumors were detected more frequently during urologic work-up of hormone disorders (*p* = 0.001). Preoperative tumor size was a strong predictor of malignancy (area under the curve (AUC) = 0.726; *p* < 0.001). According to the Youden index, the best cutoff to predict tumor dignity was 13.5 mm, resulting in a sensitivity and specificity of 53% and 85%, respectively. No cases of local recurrence or distant metastasis were confirmed after a median follow-up of 42 months. **Conclusion:** Our findings are consistent with previous reports, supporting an OSS approach in small testicular tumors whenever possible. Most tumors ≤ 20 mm were benign, and in the case of malignancy, OSS with FSE and consecutive orchiectomy is oncologically safe due to the high concordance rate of FSE and final histology, thus preventing a two-stage procedure.

## 1. Introduction

Testicular cancer is the most common malignant disease in young men, accounting for 1% of male neoplasms and 5% of urological tumors, with a mean incidence of 4 to 8 in 100,000 males per year [1]. Established guidelines recommend that the standard of care should consist of radical orchiectomy if distinct features of malignancy are present and given a normal contralateral testis [2,3]. Current recommendations for organ-sparing surgery (OSS) are confined to rare conditions such as synchronous bilateral tumors or tumors in solitary testes occupying <30% of the total testicular volume, if associated with normal findings in computed-tomography (CT) scans, a normal hormonal status, and negativity for preoperative tumor markers [3]. With high-frequency ultrasonography (US) today being widely used for scrotal examinations, nonpalpable small testicular tumors are detected in increasing numbers as incidental findings [4,5]. More than 80% of asymptomatic, nonpalpable, and marker-negative lesions smaller than 20 mm have been shown to be benign [6,7].

Against this background, radical orchiectomy is often tantamount to overtreatment and may cause infertility, psychological problems, and endocrine disorders [8,9]. Hence, it has been suggested that patients presenting with inconclusive findings should be managed by primary tumor enucleation followed by intraoperative frozen section examination (FSE), as FSE during OSS is highly concordant with final histology [10,11,12].

Even though no specific predictive biomarkers are available to confidently distinguish between benign and malignant lesions prior to surgery, imaging techniques have significantly improved over the past years. Multiparametric testicular US, combining strain elastography and contrast-enhanced US, seems to be a very promising diagnostic tool in the differentiation of benign and malignant lesions with an impressive diagnostic accuracy (sensitivity and specificity of 100% and 93%, respectively) [13]. Moreover, evidence has been provided that lesion size on preoperative US might be directly associated with malignancy, meaning that smaller tumors are more often benign [14]. Nevertheless, small testicular tumors are currently not routinely managed with a view to preserving testicular parenchyma, even though OSS is an established and safe approach [7], which also has been confirmed in previous studies at our department [15,16]. Additionally, there is no clear definition of ‘small’ tumors concerning the exact tumor diameter in which a primary OSS approach should be performed in current guidelines.

These considerations prompted us to evaluate the OSS approach for small testicular tumors, defined as ≤20 mm, in a retrospective analysis of a 15-year single-center testicular cancer database. We will focus on the diagnostic accuracy of preoperative US in determining lesion size, and in predicting malignancy, against the gold standard of postoperative histopathological assessment.

## 2. Experimental Section

### 2.1. Study Design and Case Selection

We retrospectively reviewed our medical records of all patients who underwent OSS for small testicular tumors at our center for the past 15 years. Based on our institutional practice, all patients with small testicular masses ≤20 mm, peripherally located, marker-negative, and unsuspicious according to preoperative CT scan underwent a primary OSS approach.

### 2.2. Preoperative Examinations and Follow-Up

All patients had undergone a preoperative assessment including a clinical physical examination, bilateral testicular US, CT scanning (pelvis, abdomen, chest), and an evaluation of tumor markers such as alpha-fetoprotein (AFP), beta-human chorionic gonadotropin (β-HCG), and lactate dehydrogenase (LDH) and hormone status (luteinizing hormone (LH), follicle stimulating hormone (FSH), testosterone, estradiol, and prolactin) according to the current European Association of Urology (EAU) guidelines [17]. Preoperative cryopreservation was offered to every fertile patient before surgery. Follow-up examinations after surgery were performed according to our institutional practice at our outpatient uro-oncology department. The detailed work-up of follow-up visits has already been described previously [16,18].

### 2.3. Surgical Approach of OSS

All testes were explored through the inguinal approach, in 9 (9.1%) of 99 cases verifiably associated with spermatic cord clamping and ischemia [7,19]. Details about the surgical technique of OSS without ischemia are described in detail in previous studies [14,16]. Briefly summarized, once the testis had been extracted from the scrotum, the tunica vaginalis was opened, and the tumor was identified by palpation and/or intraoperative ultrasonography. Unless the tumor was palpable, a needle was placed next to the lesion under ultrasound guidance. Then the tunica albuginea was incised at the point closest to the tumor, and enucleation was performed for frozen section examination. In addition, multiple biopsies of the surrounding parenchyma and tumor bed were obtained to exclude any germ cell neoplasia in situ (GCNIS). Radical orchiectomy was performed if frozen section examination confirmed malignancy or, alternatively, the organ-sparing procedure was completed if it was found to be benign.

### 2.4. Outcome Measures

The size (diameter) of each testicular lesion was determined preoperatively by an expert uroradiologist based on US images, using a high-frequency linear-array transducer and one of various ultrasound scanners that have been used for this purpose at our center over the past 15 years. Preoperative US evaluation included gray-scale US, color Doppler US, strain elastography, and contrast-enhanced US in 66 of 89 (74.2%) patients. Uropathologists at our center evaluated all surgical specimens postoperatively for definitive histology, which constitutes the gold standard of diagnosing malignant or benign tumors.

### 2.5. Data Analysis

Statistical operations were performed using IBM SPSS Statistics 25 (IBM, Chicago, IL, USA), with *p*-values ≤ 0.05 (two-tailed) considered to be significant. All data, except for descriptive patient statistics, were analyzed at the treatment level. Quantitative values were compared to postoperative histopathological findings by (nonparametric) Mann-Whitney U tests or (parametric) Student’s *t-*tests, and categorical variables were compared using chi-square or Fisher’s exact tests, as appropriate. Receiver operating characteristic (ROC) analyses for preoperative tumor size were fitted to determine the ‘area under the curve’ (AUC) and the Youden index for the best cutoff between benign and malignant, comparing the data to the histopathological findings to calculate sensitivity, specificity, and positive and negative predictive values (PPV, NPV). Ultrasonographic size was plotted in a linear regression model against histopathological size to assess the precision of the former based on the latter, along with a mixed model to account for clustering at the patient level.

## 3. Results

### 3.1. Baseline Characteristics

A total of 89 patients with testicular lesions were enrolled in this retrospective study. Ten (11.2%) of the patients had been treated for synchronous bilateral tumors, resulting in 99 enucleation procedures. Overall baseline characteristics stratified by dignity are shown in Table 1. Patients with benign disease were significantly older than those with malignant disease (mean: 41.1 vs. 32.9 years, respectively; *p* = 0.026). In addition, testicular lesions of patients with benign histology were detected more frequently during urologic work-up of hormone disorders (e.g., gynecomastia, hypogonadism, or infertility) compared to patients with malignant tumors (19.3% vs. 0%; *p* = 0.001) (Table 2). Although patients with malignant tumors presented more with palpable lesions (40.7%) compared with benign tumors (25%) during self-examination, this finding was not statistically significant (*p* = 0.138) (Table 2). Pre- and postoperative hormone status is presented in Supplementary Table S1.

### 3.2. Surgical Outcomes

The mean (± *SD*) duration of surgery was 61.6 (±20.2) minutes. Based on the final histology, 67 (67.7%) of the 99 lesions were benign and 32 (32.3%) were classified as malignant (Table 2). The frozen section histology yielded the same results as the final histology in 97 of 99 cases, resulting in a high concordance rate of 98% by FSE. Nevertheless, the remaining two patients, who had tumors that had been initially identified as ‘benign’ during FSE required second intervention with radical orchiectomy, as final histology confirmed seminoma. Concerning these two cases of seminoma, the preoperative lesion sizes were 18 mm and 7 mm, the patients were 30 and 32 years old, and both presented with suspicious findings on palpation. Thus, FSE involved a 2% rate of false-negative results. Despite one case with testicular hematoma (conservative management), no intraoperative or postoperative complications were detected. After a median follow-up of 42 months, no local recurrence or distant metastases was observed during the follow-up.

### 3.3. Tumor Size as Predictive Marker

**Figure 1:**
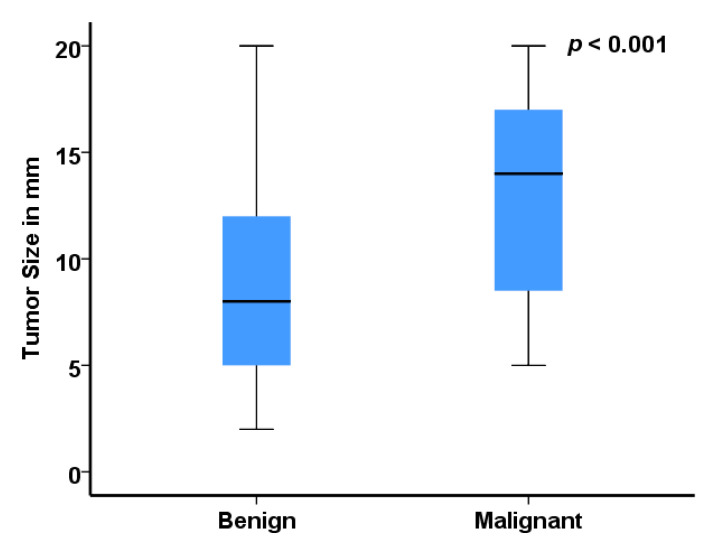
Bar graph illustrating the association between preoperative ultrasonographic results for lesion size and postoperative histopathological results for benign versus malignant tumors. Data represent mean ± *SD*.

Preoperative US results for lesion size were available for all 99 cases and yielded a median size of 10.0 mm (IQR: 6.0–15.0). Although we observed a stable trend towards OSS procedures over the last 15 years at our institution, the percentage of benign tumors per year increased due to improved imaging techniques with higher detection rate of small tumors (Supplementary Figure S1). As shown in Figure 1 and Table 2, malignant tumors were significantly larger than benign lesions (median: 14 vs. 8.0 mm; *p* < 0.001). Nevertheless, 32 (32.3%) of 99 testicular lesions ≤ 20 mm were diagnosed as malignant, and 37.5% (*n* = 12) of them were ≤10 mm (Table 3).

**Figure 2:**
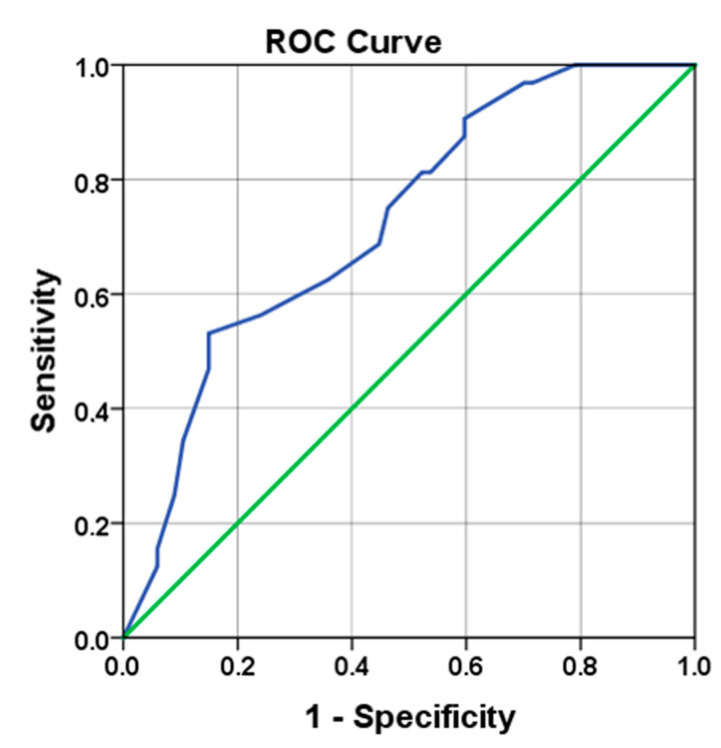
Receiver operating characteristic (ROC) curve indicating the value of ultrasonographic lesion size in predicting malignancy.

The ROC curve in Figure 2 yielded an AUC of 0.726 (95% CI: 0.623–0.828) to predict malignancy (*p* = 0.000). The best cutoff (highest Youden index score) was a lesion diameter of 13.5 mm (53% sensitivity, 85% specificity, 63% PPV, 79% NPV).

### 3.4. Final Histopathological Findings

Table 4 summarizes the final histopathological findings for all 99 testicular lesions. A total of 32 malignant neoplasms (32.3%) were identified. Of them, 22 (68.8%) cases were pure seminoma, eight (25%) cases were pure teratoma, and only two (6.2%) were classified as mixed germ cell tumors (embryonal carcinoma with small teratoma components). Concerning seminoma patients (*n* = 22), four patients (18.2%) received adjuvant treatment with one cycle of carboplatin (area under curve (AUC = 7) and ten patients (45.5%) received two cycles of carboplatin (AUC = 5). The other 67 lesions (67.7%) were defined as benign, the most common diagnoses being Leydig cell tumor or hyperplasia in 32 (47.8%) of 67 cases, followed by pseudotumors and dermoid cysts, each accounting for 10 cases (29.8%).

**Figure 3:**
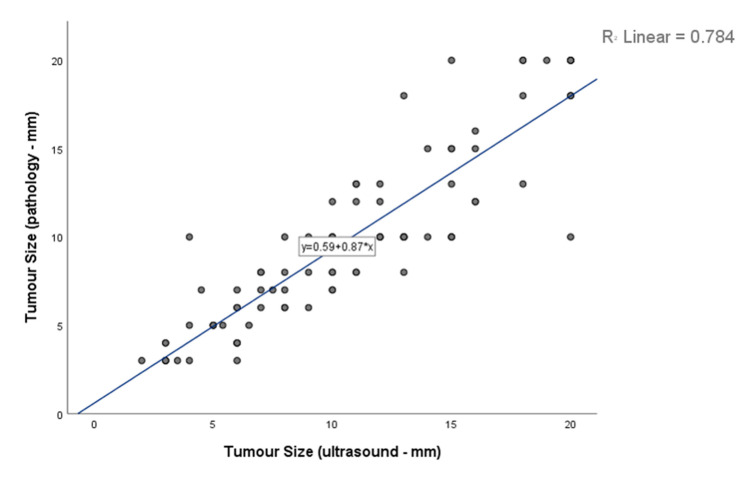
Linear regression of ultrasonographic lesion size plotted against histopathological lesion size, indicating that the former was a strong predictor of the latter (*p* < 0.001).

Consistent with the results from a mixed model accounting for clustering at the patient level (β = 0.864; 95% CI: 0.833–0.896), the linear regression in Figure 3 shows that preoperative lesion size was a strong predictor of histopathological size, with each additional millimeter of the former entailing a 0.87 mm (95% CI: 0.770–0.970) increase of the latter.

## 4. Discussion

OSS with tumor enucleation and intraoperative FSE is increasingly used for guidance in treating patients with inconclusive testicular findings [3].

Using this approach to treat small tumors is currently still not the standard of care, even though up to 80% of all nonpalpable testicular tumors ≤ 20 mm in size are benign [6,7]. On the other hand, radical surgery should be avoided whenever possible to preserve endocrine function and to minimize late-onset hypogonadism and psychological sequelae [8,9].

However, the practicability of OSS may be based on FSE reliability in the diagnosis of testicular tumors. Previous studies confirmed that FSE correlates well with final histopathological diagnosis of testicular masses, with a high specificity and sensitivity between 90–100% and 95–100%, respectively [11,12,20]. Although FSE correctly identified all nonmalignant lesions, a small failure rate of 3.5% in identifying malignant lesions was described in the study of Silverio et al. [20]. In the present work, we detected similar results with a high concordance rate of 98% by FSE, but also a 2% false-negative rate. According to these findings, patients should be informed about the possible risk of a second intervention with orchidectomy in case of incorrect FSE.

Today’s widespread use of advanced high-resolution ultrasonography is associated with small testicular lesions being detected in greater numbers, with many of them turning out to be benign [4,5]. This fact was also confirmed at our institution, where despite a stable rate of OSS procedures over the past 15 years, the percentage of benign tumors increased due to improved imaging techniques with a higher detection rate of small tumors (Supplementary Figure S1). Sonographic methods, or even magnetic resonance imaging, cannot possibly distinguish between malignant and benign testicular tumors but are perfectly capable of disclosing the accurate size of a lesion prior to surgery [21,22]. The regression analysis in Figure 3 illustrates this diagnostic quality of preoperative ultrasonography at our center, plotting the lesion measurements thus obtained against the dimensions verified postoperatively by histopathology. As expected, preoperative sonographic lesion size turned out to be a strong predictor of pathological tumor size (*p* < 0.001).

While a consistent definition of ‘small’ tumors does not exist, previous definitions have varied between 10 and 25 mm [3,23,24]. Actually, the patients involved in this study had testicular tumors ≤ 20 mm in diameter. Gentile et al. [14], who also examined patients with testicular masses less than 20 mm, found that the smaller a lesion, the less likely it was to be malignant, and reported a best cutoff of 8.5 mm [14]. At 13.5 mm, we report a considerably higher best cutoff for lesion diameter based on our series of 99 enucleation procedures (53% sensitivity, 85% specificity, 63% PPV, 79% NPV). Moreover, we found 21.8% of all lesions with a size ≤ 10 mm (12 of 55) to be malignant, which is a considerably higher rate than the 10% reported by Gentile et al. [14]. Applying the confirmed cutoff of 8.5 mm by Gentile et al. [14], we obtained similar results concerning the diagnostic accuracy (75% sensitivity, 54% specificity, 44% PPV, 82% NPV). Thus, our results are in line with the findings by Gentile et al. [14], supporting the fact that tumoral lesion size may be a predictor of malignancy. In addition, our study adds to an increasing body of evidence that OSS is an oncologically safe approach to small testicular tumors [10,11,14,16,23,25].

On the other hand, it would certainly be inappropriate to regard the predictive merits of tumor size as an absolute, and our series naturally did include benign tumors larger and malignant tumor smaller than the 13.5 mm cutoff we obtained. In detail, 21 of our patients with very small lesions ≤ 5 mm included just one single malignancy (taking the form of a post-pubertal type teratoma). Bieniek et al. [26] reported a growth rate of 0.01 mm per year for very small testicular lesions (mean size: 4.14 mm) without any apparent implications for disease progression [26]. Against this background, our results are consistent with previous suggestions that a surveillance strategy could be adopted in patients with “very small” testicular lesions ≤ 5 mm, using serial ultrasound monitoring but not resorting to surgical intervention unless the tumor is actually found to grow [12,26].

According to the current EAU guidelines, the standard surgical approach to testicular tumors is inguinal exploration with exteriorization of the testis within its tunics [3]. Whether warm or cold ischemia may be beneficial continues to be unclear. While spermatic cord clamping has been used to prevent tumor seeding, there may be a risk of long-term side effects, such as vessel injury with endocrinological sequelae or obstruction of the vas deferens [16]. Protracted suppression of the blood supply is known to inflict irreversible damage to testes [27]. Apparently, there is a major point in not exceeding 30 min of surgery, given the demonstration by Miller et al. [28] that longer periods of warm ischemia will involve morphological changes in Sertoli cells. As shown in Table 2, only nine procedures (9.1%) in our study did include clamping, while 87 (88.9%) did not. Based on malignant tumors, 27 (84.4%) of 32 enucleations were performed without spermatic cord clamping. Given incomplete records on hormonal and seminal profiles, we are unable to conclusively judge the implications of such clamping at this time.

Limitations of this study notably include its retrospective nature and moderate sample size. Moreover, worth mentioning are differences in the follow-up periods between malignant and benign cases, in the types of surgery performed (irrespective of spermatic cord clamping), and between the uropathologists examining the biopsies. Although these examinations took place in the same regional center, an effect of different experience levels on the sensitivity of frozen section results cannot be completely ruled out [11]. However, regardless of surgical techniques, preoperative tumors sizes, or benign versus malignant outcomes, and reiterating the caveat of significantly shorter follow-ups among the benign cases, no cases of systemic progression or recurrence were noted.

In conclusion, a primary OSS approach seems to be justified in small (≤20 mm), marker-negative clinical stage I testicular tumors whenever possible, as (i) approximately two-thirds of all lesions were classified as benign at final histology, and (ii) based on the high concordance rate of FSE with final histology, malignant tumors were detected correctly in 93.8% by FSE, thus preventing a two-stage procedure to complete radical orchiectomy. Therefore, a multistep procedure, which includes tumor enucleation, FSE followed by either OSS or radical orchiectomy based on FSE histology, provides an oncologically safe and practicable surgical management in the therapy of patients with small testicular tumors. Although the false-negative rates of FSE are very low, patients should be informed about the risk of second intervention. Prospective studies are needed to establish OSS in the management of testicular cancer and to expand this step-by-step approach by finding the best surveillance strategy also for patients with “very small” tumors ≤ 5 mm in diameter.

## Figures and Tables

**Table 1:** Summary of baseline characteristics at the patient level.

	All Tumors		Benign Tumors		Malignant Tumors		*p*-Value
	Value	%, *SD*, IQR	Value	%, *SD*, IQR	Value	%, *SD*, IQR	
Number of patients (*n*)	89		60		29		0.581 (exact)
Unilaterally treated (*n*)	79	88.8%	53	88.3%	26	89.7%	
Bilaterally treated (*n*)	10	11.2%	7	11.7%	3	10.3%	
Age (years)							0.026
Available (*n*)	89	100%	60	100%	29	100%	
Mean ± *SD* (years)	38.4	±16.2	41.1	±16.8	32.9	±13.5	
Duration of surgery (min)							0.674
Available (*n*)	52	58.4%	40	66.7%	12	41.4%	
Mean ± *SD* (months)	61.6	±20.2	62.3	±20.7	59.4	±19.2	
Follow-up (months)							0.017
Available (*n*)	89	100%	60	100%	29	100%	
Median (IQR) (months)	42	(3.5–75.5)	32.5	(1.3–64.8)	59	(29.5–100)	

**Table 2:** Summary of data analyzed at the treatment level.

	All Tumors		Benign Tumors		Malignant Tumors		*p*-Value
	Value	%, IQR	Value	%, IQR	Value	%, IQR	
Number of treatments (*n*)	99		67		32		
Ultrasonographic size (mm)							<0.001
Available (*n*)	99	100%	67	100%	32	100%	
Median (IQR) (mm)	10	(6.0–15.0)	8.0	(5–10.0)	14	(8.3–17.5)	
Histopathological size (mm)							0.018
Available (*n*)	85	85.9%	56	56.6%	29	90.6%	
Median (IQR) (mm)	10	(6.0–13.0)	8.0	(5.3–10.0)	10.0	(8.0–15.0)	
Palpation (*n*)							0.138
Available (*n*)	87	87.9%	60	89.6%	27	84.4%	
Positive (*n*)	26	29.9%	15	25.0%	11	40.7%	
Ischemia (*n*)							0.461 (exact)
Available (*n*)	97	98.0%	66	98.5%	31	96.9%	
Yes (*n*)	9	9.3%	5	7.6%	4	12.9%	
Clinical presentation (*n*)							0.001 (exact)
Available (*n*)	82	82.8%	57	85.1%	25	78.1%	
Lump/swelling (*n*)	23	28.0%	16	28.1%	7	28.0%	
Pain (*n*)	11	13.4%	7	12.3%	4	16.0%	
Incidental (*n*)	31	37.8%	23	40.4%	8	32.0%	
Inf/hyp/gyn ^1^ (*n*)	11	13.4%	11	19.3%	0	0%	
Oncologic follow-up (*n*)	6	7.3%	0	0%	6	24.0%	

^1^ Infertility/hypogonadism/gynecomastia.

**Table 3:** Testicular tumors stratified by tumor size.

Tumor Size (mm)	Overall (*n* = 99)	Benign (*n* = 67)	Malignant (*n* = 32)
≤5 mm, *n* (%)	21	20 (29.9)	1 (3.1)
>5 mm and ≤10 mm, *n* (%)	34	23 (34.3)	11 (34.4)
>10 mm and ≤15 mm, *n* (%)	26	17 (25.4)	9 (28.1)
>15 mm and ≤20 mm, *n* (%)	18	7 (10.4)	11 (34.4)

**Table 4:** Histopathological classification of the testicular tumors analyzed in this study.

Malignant and Benign Lesions	*n*	%
Seminomatous germ-cell tumor	22	22.2
Nonseminomatous: pure teratoma	8	8.1
Nonseminomatous: mixed germ cell ^1^	2	2.0
Leydig cell tumor or hyperplasia	32	32.3
Sertoli cell tumor	4	4.0
Fibrotic pseudotumor	10	10.1
Adenomatoid tumor	2	2.0
Dermoid cyst	10	10.1
Cystic lesion	3	3.0
Hemangioma	4	4.0
Splenogonadal fusion	1	1.0
Leiomyoma	1	1.0
Total	99	100

^1^ Teratoma/embryonal carcinoma.

## Supplementary Materials

The following are available online at https://www.mdpi.com/2077-0383/9/9/2911/s1, Figure S1: Percentage of malignant and benign tumors from organ-sparing surgery (OSS) approaches (Medical University of Innsbruck) over the past 15 years, Table S1: Hormonal profiles of 60 patients finally treated with OSS.

## Abbreviations

AFP	alpha-fetoprotein
AUC	area under the curve
CT	computed tomography
EAU	European Association of Urology
FSE	frozen section examination
GCNIS	germ cell neoplasia in situ
β-HCG	beta-human chorionic gonadotropin
IQR	interquartile ranges
IV	intravenous
LDH	lactate dehydrogenase
NPV	negative predictive values
OSS	organ-sparing surgery
PPV	positive predictive values
ROC	receiver operating characteristic
US	ultrasonography
