# Supplementary material for: Organ-Sparing Surgery in Testicular Tumor: Is This the Right Approach for Lesions ≤ 20 mm?
Source: J Clin Med. 2020 Sep 9;9(9):2911. doi: 10.3390/jcm9092911 (PMC7565605; doi:10.3390/jcm9092911)
Supplement: Supplementary file 1 [file jcm-09-02911-s001.pdf]

Supplementary Materials:

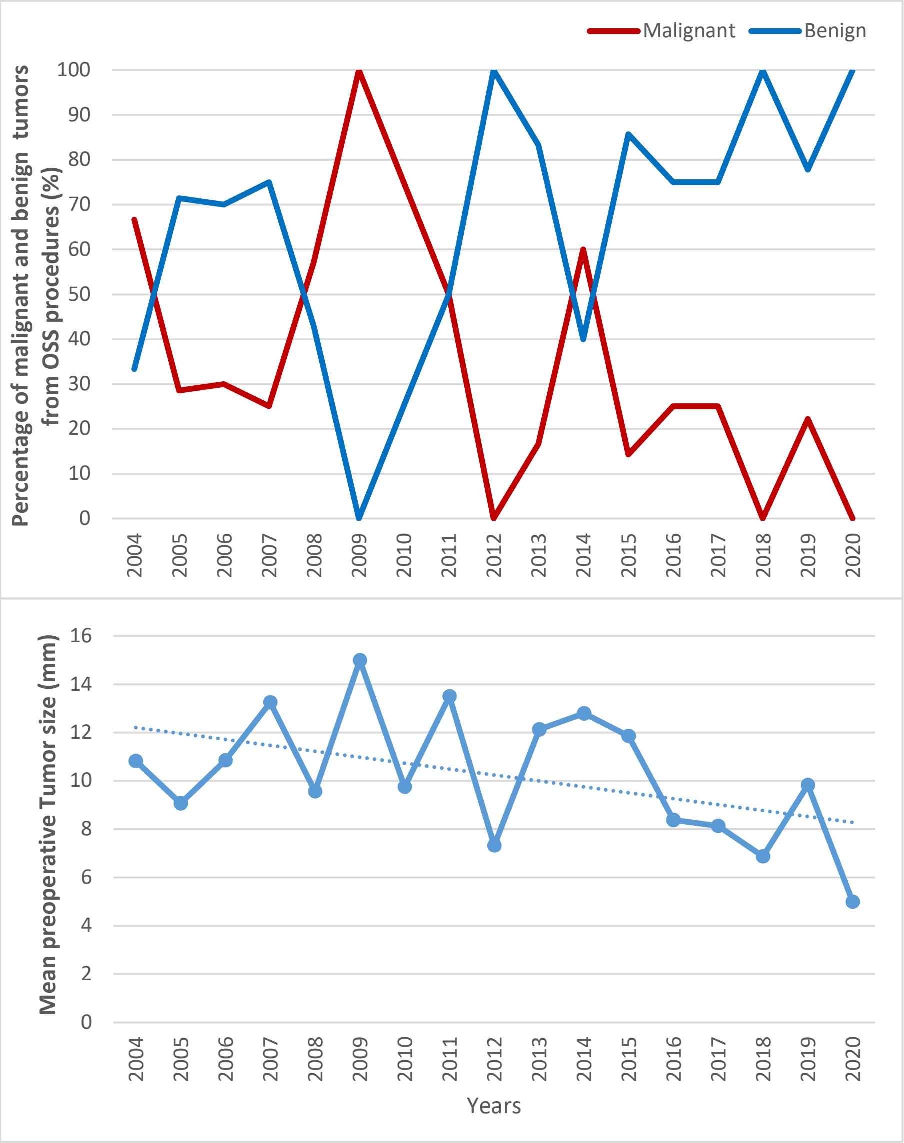

Supplementary Figure 1. Percentage of malignant and benign tumors from organ-sparing surgery (OSS) approaches (Medical University of Innsbruck) over the past 15 years. Despite the stable trend toward enucleation procedures, the incidence of benign tumors has increased over the past few years due to improved imaging techniques with increased detection of small tumors.

Supplementary Table 1. Hormonal profiles of 60 patients finally treated with OSS.

	Before surgery		After surgery		*p*-value
	**Value**	%, *SD*, IQR	Value	%, *SD*, IQR	
**LH**					-
Available (*n*)	40/60	66.7%	0/60	-	
Mean ± *SD*	5,68	± 5.08, (1–22.1)	-	-	
**FSH**					0.133
Available (*n*)	40/60	66.7%	10/60	16.7%	
Mean ± *SD*	12.63	± 13.92, (1.5–56)	19.11	± 13.23, (2.6–44.4)	
**Testosterone**					0.155
Available (*n*)	41/60	68.3%	10/60	16.7%	
Mean ± *SD*	4.22	± 1.51, (1.1–7.7)	3.45	± 1.52, (0.87–5.69)	
**Estradiol**					0.809
Available (*n*)	39/60	65%	10/60	16.7%	
Mean ± *SD*	27.51	± 9.69 (13–50)	26.7	± 8.22, (13–41)	
**Prolactin**					0.903
Available (*n*)	33/60	55%	10/60	16.7%	
Mean ± *SD*	9.13	± 4.72, (3.7–23.5)	9.35	± 5.69, (3.2–22.6)	

Sex-specific reference values: 0.8–7.6 U/L for luteinizing hormone (LH), 1.6–20.4 U/L for follicle stimulating hormone (FSH), 1.70–4.90 μg/L for testosterone, 11–43 ng/L for estradiol, and 2.5–17.0 μg/L for prolactin.
